# Effects of Short-Term Cognitive Remediation on Cognitive Dysfunction in Partially or Fully Remitted Individuals with Bipolar Disorder: Results of a Randomised Controlled Trial

**DOI:** 10.1371/journal.pone.0127955

**Published:** 2015-06-12

**Authors:** Kirsa M. Demant, Maj Vinberg, Lars V. Kessing, Kamilla W. Miskowiak

**Affiliations:** Copenhagen Affective Disorder Clinic, Psychiatric Centre Copenhagen, Copenhagen University Hospital, Rigshospitalet, Copenhagen, Denmark; The University of Queensland, AUSTRALIA

## Abstract

**Introduction:**

Cognitive dysfunction is common in bipolar disorder (BD) but is not sufficiently addressed by current treatments. Cognitive remediation (CR) may improve cognitive function in schizophrenia but no randomised controlled trial has investigated this intervention in BD. The present study aimed to investigate the effects of CR on persistent cognitive dysfunction in BD.

**Method:**

Patients with BD in partial remission with cognitive complaints were randomised to 12 weeks group-based CR (n=23) or standard treatment (ST) (n=23). Outcomes were improved verbal memory (primary), sustained attention, executive and psychosocial function (secondary) and additional measures of cognitive and psychosocial function (tertiary). Participants were assessed at baseline and weeks 12 and 26.

**Results:**

Of the 46 randomised participants five dropped out and one was excluded after baseline. CR (n=18) had no effect on primary or secondary measures of cognitive or psychosocial function compared with ST (n=22). However, CR improved subjective sharpness at week 12, and quality of life and verbal fluency at week 26 follow-up (tertiary outcomes). Although the trial turned out to have suboptimal statistical power for the primary outcome analysis, calculation of the 95% confidence interval showed that it was highly unlikely that an increase in sample size would have rendered any beneficial effects of CR vs. ST on the verbal memory.

**Conclusions:**

Short-term group-based CR did not seem to improve overall cognitive or psychosocial function in individuals with BD in full or partial remission. The present findings suggest that that longer-term, more intensive and individualised CR may be necessary to improve cognition in BD.

**Trial Registration:**

ClinicalTrials.gov NCT01457235

## Introduction

Cognitive dysfunction is a core feature in a large proportion of individuals with bipolar disorder (BD) [1–3]. Pervasive deficits during periods of remission have been shown particularly in verbal memory, sustained attention, executive function [4,5] and social cognition [6,7]. It has been estimated that 30–60% of individuals with BD experience such trait-related cognitive dysfunction and that this is a key mediator of their occupational and psychosocial difficulties [8–10] and reduced quality of life [11]. Nevertheless, there is no well-established pharmacological or psychological treatment for cognitive deficits in BD. Clinical studies of new pharmacological treatments to target cognitive deficits in BD have revealed no convincing effects [12,13]. Only one explorative study from our group showed substantial mood-independent cognitive improvement in response to erythropoietin (EPO) over placebo in partially remitted individuals with BD [14]. A new psychological treatment, cognitive remediation (CR), aims to improve cognitive function, compensational skills and coping. A meta-analysis of 40 studies of CR in schizophrenia demonstrated that CR produces cognitive and functional improvement in this patient group (Effect size = 0.45, 95% CI 0.31–0.59) [15]. However, the effects of CR are less evident in BD; a recent study investigated effects of functional remediation (FR), an intervention similar to CR but mainly targeting psychosocial function [16]. Although FR improved psychosocial function compared with standard care (small effect size; d’ = 0.3), the FR group did not differ from a group receiving psychoeducation (PE) and there were no cognitive benefits of FR over PE or standard care [16]. Three studies have investigated the effects of CR in affective disorders including participants with BD: an open trial with 18 bipolar individuals and no control group [17], a preliminary study with 14 unipolar and two bipolar individuals allocated (1:1) to either CR or waiting list [18], and a proof of principle study with 24 unipolar individuals allocated (1:1) to either CR or control treatment and, additionally, 22 healthy controls [19]. Overall, these studies found some evidence for improvement on verbal memory, attention and executive function in response to CR. However, the findings on the effects in BD can only be considered preliminary since the studies were not randomised, used small samples, in one case had no control group and in two cases included mixed groups of unipolar and bipolar individuals. To date no randomised controlled trial has investigated the effects of CR on cognitive dysfunction in BD. The present trial therefore aimed to investigate the effects of group-based CR on cognitive dysfunction in individuals with BD who experienced cognitive difficulties despite being in partial or full remission. Given the above preliminary evidence for beneficial effects of CR in affective disorders [17–19] and evidence for trait-related deficits in these cognitive domains in BD [4,5], we hypothesised that CR would improve cognitive and psychosocial function in comparison with standard treatment (ST). In this trial, we chose cognitive function (over functional outcome) as the primary study outcome because cognitive dysfunction is an important mediator of psychosocial dysfunction [20,21] and because no available treatments target cognitive dysfunction in BD.

## Method

### Study design

The trial design has been published in full [22]. Participants were randomized (1:1) to receive either 12 weeks add-on group-based CR or ST in an evaluator-blind, between-groups design.

### Participants

Participants were recruited from the Copenhagen Affective Disorder Clinic, Psychiatric Centre Copenhagen, Copenhagen, Denmark, community psychiatric centres, specialists in psychiatry in private clinics and general practices. Eligible participants had an ICD-10 diagnosis of BD according to the Schedule for Clinical Assessment in Neuropsychiatry (SCAN) [23], were aged 18 to 50 years, had subjective cognitive difficulties according to the Massachusetts General Hospital Cognitive and Physical Functioning Questionnaire (CPFQ) [24] (score >4 on > 2 domains) and were in full or partial remission (Hamilton Depression Rating Scale—17 items (HDRS-17) [25] and Young Mania Rating Scale (YMRS) [26] scores of ≤14). The inclusion of partially (in addition to fully) remitted patients aimed to ensure a sufficient sample size and was informed by evidence suggesting that residual affective symptoms have no major effects on objective cognitive function [27]. Exclusion criteria were a diagnosis of schizophrenia or schizoaffective disorder, significant suicide risk, ECT-treatment within the past three months and current substance or alcohol abuse. Participants were permitted to take antidepressant, lithium, antipsychotic medication and benzodiazepines (corresponding to < 22.5 mg Oxazepam daily).

### Interventions

Participants randomised to the CR group received CR in addition to standard treatment. CR was conducted in a group-setting in weekly sessions of two hours over 12 weeks followed by a booster session four weeks after treatment completion. The rationale behind choosing a group-setting over individual treatment was that being in a group with others who experience the same problems, in this case cognitive difficulties, tends to be a motivational factor. Also, group-settings are more cost-effective than individual treatment (thus enhancing the chances of potential clinical implementation of this treatment). Finally, short-term PE in a group-setting has been shown to have beneficial effects in BD [16]. The rationale for choosing a short-term treatment of 12 weekly sessions was that CR treatment durations vary across studies and there are no indications that a longer duration will result in greater cognitive improvement. Therefore, it seemed reasonable to investigate the effects of a short-term intervention given the greater feasibility and cost-effectiveness of a short-term versus a longer-term treatment. The 12 sessions were divided into four topics: the first two sessions were an introduction to cognitive function and dysfunction and cognitive remediation. Sessions three to five involved training of and tackling difficulties with attention and concentration; memory and learning were addressed in sessions six to eight; the final four sessions targeted executive function in everyday life. Each CR session consisted of three main components: PE and awareness of cognitive dysfunction in BD for approximately 30 minutes, training of compensatory and strategies for cognitive dysfunction for about one hour and the remaining 30 minutes was spent on computer-assisted cognitive training using RehaCom software [28]. Computer exercises and compensatory and adaptive strategies targeted memory, attention and executive function and were highly ecologically valid as they focused on real-life cognitive demands such as memorising short articles or peoples’ faces and names, concentrating whilst reading, planning activities, shopping, counting, concentrating on more than one task at a time etc. Additionally, participants were encouraged to do homework on a daily basis, mainly consisting of computerised exercises but also mindfulness exercises and practising reading strategies using a short textbook on cognitive dysfunction in BD. The manual used in the present trial is not published, however, a more detailed description of the CR programme including examples of compensatory and adaptive strategies can be seen in the published study protocol [22].

Participants randomised to ST continued their current treatment: 10 participants (45%) were treated with combined psychopharmacological treatment and 16-weeks group-based psychoeducation at the Copenhagen Affective Disorder Clinic [29]; eight participants (36%) continued psychopharmacological treatment at private psychiatrists; three participants (14%) were treated at local community mental health centres who typically offer combined psychopharmacological treatment and programs with shorter group-based PE; and one participant (5%) continued psychopharmacological treatment with a general practitioner. Those who had received PE had all finished the PE course prior to the inclusion in the present trial. In contrast with the intervention group, ST did not involve any specific cognitive training.

### Outcomes

The primary outcome was change in verbal memory measured with the Rey Auditory Verbal Learning Test (RAVLT) [30,31] from pre- to post treatment (week 12). The rationale behind choosing RAVLT as the primary outcome was (i) that BD individuals tend to show trait-related deficits on this test [32,33]; (ii) RAVLT is a standardised, widely used and valid measure of verbal memory function [34]; (iii) verbal memory can be improved with CR according to studies in schizophrenia [35–37]; and (iiii) verbal memory correlates highly with psychosocial function [21] which makes it a particularly clinically relevant outcome. Secondary outcomes were sustained attention, executive function and psychosocial function as measured with the Rapid Visual Information Processing (RVP) from Cambridge Cognition (CANTAB), Part B of the Trail Making Test (TMT-B) [38] and the Functional Assessment Short Test (FAST) [39], respectively. Tertiary outcomes were additional measures of attention (Repeatable Battery for the Assessment of Neuropsychological Status (RBANS) coding [40]), memory (Delayed Matching to Sample (DMS) and Spatial Working Memory (SWM) from CANTAB), executive function (WAIS-III Letter-Number Sequencing [41]; Verbal Fluency [42]; RBANS digit span [40]), psychomotor speed (TMT-A) [38], reaction time (Simple Reaction Time (SRT) from CANTAB) and facial expression recognition (Facial Expression Recognition Task (FERT) from the Oxford Emotional Test Battery (P1 Vital; Oxford)) and, additionally, self-reported cognitive and psychosocial function, stress, coping strategies, depressive symptoms and quality of life as measured with CPFQ [24]; Cognitive Failures Questionnaire (CFQ) [43]; WHO Quality of life BREF (WHOQOL-Bref) [44]; Cohen’s Perceived Stress Scale (PSS) [45]; European Quality of Life—5 Dimensions—3 Levels (EQ-5D-3L) [46]; Beck Depression Inventory (BDI) [47] and Work and Social Adjustment Scale (WSAS) [48]. We hypothesized improvement of these outcome measures from baseline to week 12 for participants randomised to CR vs. ST.

### Randomisation and blinding

Pharma Consulting Group [49] performed the randomisation of participants with stratification for age (<35 years) and years of education (<15 years). Study personnel involved in the evaluation of outcomes were blinded to treatment allocation. In cases where the evaluator became aware of treatment allocation a different, blinded evaluator would carry out further assessments. Blinding was maintained throughout the study, data management, outcome assessment and data analysis.

### Sample size calculation

Sample and statistical power was calculated before trial start by Pharma Consulting Group A/S) using nQuery Advisor 5.0 software. The primary outcome was *change* in verbal memory measured with RAVLT from baseline to week 12 between CR and ST groups (i.e. two time points and two groups). A study had demonstrated that the average California Verbal Learning Test (CVLT; a verbal learning test equivalent to the RAVLT) total recall score for patients with remitted bipolar disorder is 52.0 whilst healthy controls matched on age was 60.7 (out of maximum 75) [50]. The original assumption, on which the sample size calculation was based, was therefore that a clinically relevant difference between CR and ST groups in the *change* in RAVLT total recall would be 4 points (half-way to normal) with a standard deviation of 4 points of RAVLT *change* scores. Based on this, it was calculated that sample size of n = 40 patients (n = 20 per group) would achieve a statistical power of 86% to demonstrate a clinically relevant verbal memory improvement with CR versus ST from baseline to week 12 (see the original methods paper; [22]). The original sample size calculation and the statistical analysis of the primary outcome were thus essentially based on the same strategies: analysis of the differences between the two groups in the *change* in RAVLT total scores from baseline to week 12.

### Statistical analyses

All statistical analyses of behavioural data, mood ratings and questionnaires were conducted using the Statistical Package for Social Sciences. We used repeated measures analysis of covariance (ANCOVA) from baseline to week 12 with adjustment for stratification variables and for cognitive test measures also with adjustment for changes in HDRS-17 and YMRS scores as mood symptoms have been shown to correlate with cognition [51]. All participants completed the RAVLT (primary outcome) and additional neuropsychological assessments at baseline and post-treatment except from one participant who did not complete one RAVLT subtest. Thus there would have been no difference in analyses of completers vs. intention-to-treat (ITT). To investigate long-term effects of CR versus ST at week 26, we implemented a linear mixed-effects model structured as a two-level model specifying a correlation of samples within participants with adjustments similar to the procedure for ANCOVA analyses. A linear mixed-effects model takes into account missing values and intra-individual variations over time and is, therefore, an appropriate statistical method for analysing data from more than two assessment times [52]. Significant interactions over time were analysed further with simple main effect analyses. All analyses were performed as per protocol [22]. In addition, we also performed univariate analyses to adjust for significant baseline differences between groups in relation to RAVLT total recall and delayed recall. Baseline scores were included as covariates and, consequently, these results will reflect differences, if any, between groups at week 12 and 26 rather than over time. For more details see our study protocol [22].

### Ethics statement

The study was approved by The Regional Committee on Biomedical Research Ethics (protocol number H-1-2010-039), the Danish Data Protection Agency (protocol number 2010-41-4710) and was registered at clinicaltrials.gov (identifier NCT01457235). Written informed consent was obtained from all participants.

## Results

### Patient flow and characteristics

**Fig 1:**
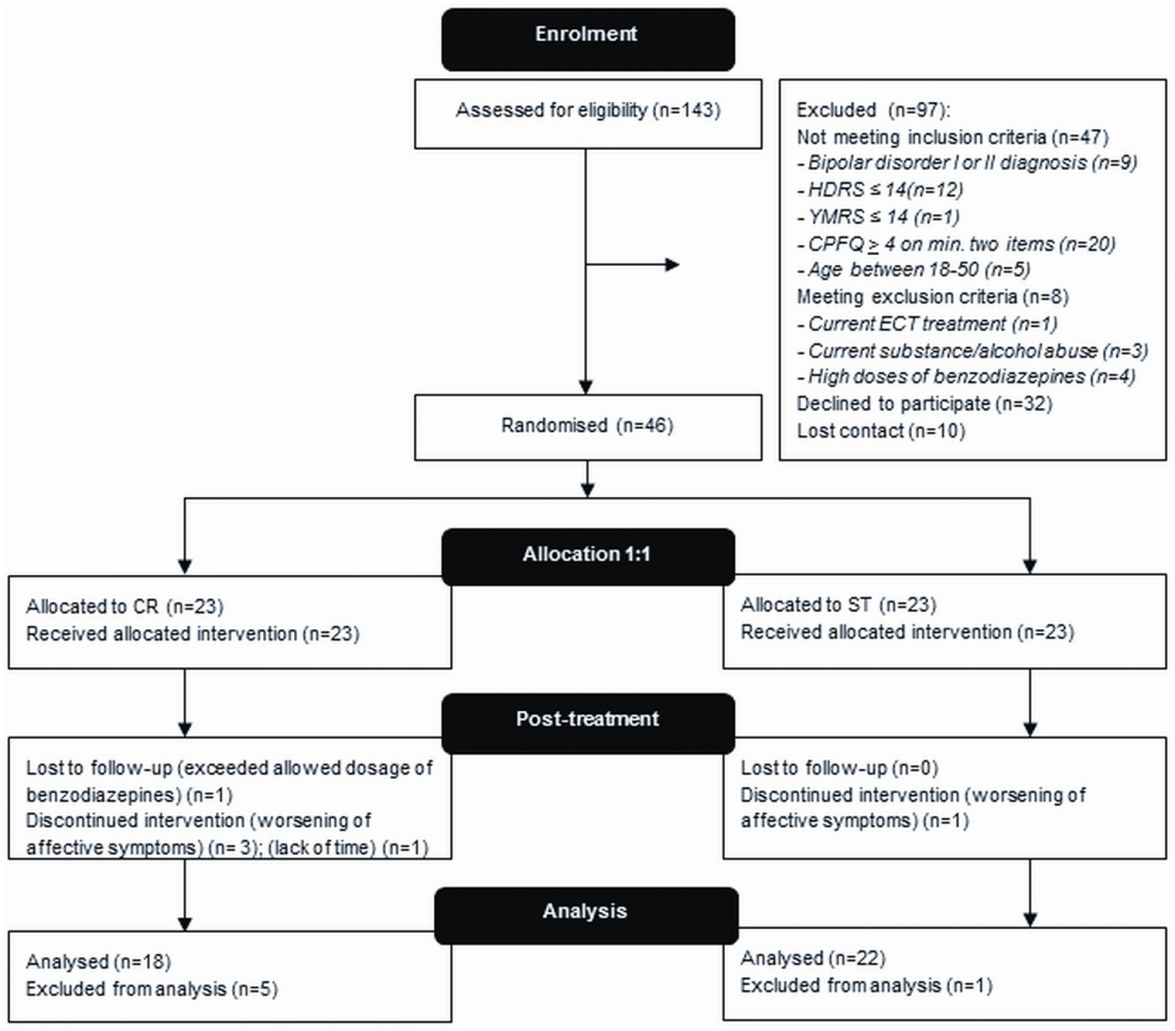
CONSORT flow-chart.

Fig 1 and Table 1 display participant flow and characteristics, respectively. 143 participants were assessed for eligibility of which 97 were excluded and a total of 46 participants were randomised to either add-on CR or ST of which six participants dropped out between baseline and week 12 (see Fig 1 for reasons). Since only baseline measures were obtained for these six participants, they could not be included in the analysis. Hence a total of 40 participants (CR n = 18; ST n = 22) were included in the analysis. As can be seen from Table 1, groups were well-matched on baseline characteristics (p-values>0.07). There were no significant differences in mood over time between groups (p-values>0.4) as measured with HDRS-17 and YMRS.

**Table 1:** Participant characteristics by treatment group at baseline.

	CR	ST
Characteristic	(n = 18)	(n = 22)
Age in years, mean (SD)	33.,9 (6.,8)	34 (7.,9)
Years of education, mean (SD)	15.,9 (2.,8)	15.,7 (3.,5)
Gender, no. Female (%)	12 (66.,7)	13 (59.,1)
Verbal intelligence (DART), mean (SD)	34.,1 (5.,7)	34.,1 (6.,1)
HDRS-17 score, mean (SD)	7.,6 (5.,7)	7.,8 (5,.9)
YMRS score, mean (SD)	3.,1 (3.,2)	2.,5 (2.,8)
CPFQ score, mean (SD)	26,.4 (4,.3)	25,.8 (4,.2)
Bipolar I diagnosis, no. (%)	13 (72.,2)	14 (63.,6)
**Bipolar disorder with psychotic features, no. (%)**	**9 (50)**	**6 (27,3)**
Number of Previous Depressive Episodes, mean (SD)	6.,2 (8.,7)	9,.5 (10,.4)
Number of Previous Manic Episodes, mean (SD)	3.,3 (6.,9)	2.,9 (8.,4)
Number of Previous Hypomanic Episodes, mean (SD)	5.,4 (5,.4)	15.,2 (22)
Medication		
*Lithium*, *no*. *(%)*	13 (72.,2)	14 (63.,6)
*Anticonvulsants*, *no*. *(%)*	14 (77,.8)	17 (77.,3)
*Antidepressants*, *no*. *(%)*	4 (22.,2)	7 (31,.8)
*Antipsychotics*, *no*. *(%)*	9 (50)	11 (50)
*Benzodiazepines*, *no*. *(%)*	2 (11.,1)	1 (4.,5)
*Number of medications*, *mean (SD)*	2.,3 (1)	2,3 (0.,8,)

Abbreviations: CR, cognitive remediation; ST, standard treatment; SD, standard deviation; DART: Danish Adult Reading Test; HDRS-17: Hamilton Depression Rating Scale 17 items; YMRS: Young Mania Rating Scale; CPFQ: Cognitive and Physical Functioning Questionnaire.

### Primary outcome

Table 2 summarises results for primary and secondary outcomes. RAVLT total recall across the five learning trials (I-V) and delayed recall showed superior baseline performance in the CR group (p-values>0.030). Baseline performance on RAVLT immediate recall and recognition was comparable across the two groups (p-values>0.069). Results showed no effects of CR over ST on total recall, delayed recall, immediate recall or recognition from baseline to week 12 (p-values>0.098) or week 26 (p-values>0.190). When adjusting for baseline differences, results still indicated no significant differences between groups on RAVLT total recall or delayed recall at week 12 (p-values>0.566) or at week 26 (p-values>0.749).

**Table 2:** Between-group differences statistics for primary and secondary outcomes.

	Baseline			Time (weeks 0–12)	Time (weeks 0–26)
	Week 0	Week 12	Week 26	Time—by group^b^	Time—by group^c^
	Mean (SD)	Mean (SD)	Mean (SD)	p value^a^	p value^a^
**RAVLT**					
*Total recall across trials (I-V) (n = 40*, *37)*					
CR	50.,94 (8.,49)	50.,39 (12.,63)	53.,35 (11.,51)	0.257	0.091 (df = 2,73)
ST	45 (9.,43)	47.,64 (8.,84)	48.,8 (8.,15)	0.566 (df = 1,33)^d^	0.749 (df = 1,30)^d^
*Recall following interference (VI) (n = 40*, *37)*					
CR	10.,89 (2)	10.,5 (2.,57)	11.,18 (2.,53)	0.646	0.200
ST	9.,32 (3.,08)	10.,27 (3.,10)	10.,8 (2.,84)	0.098 (df = 1,34)	0.190 (df = 2,72)
*Delayed recall (30 mins) (n = 40*, *37)*					
CR	10.,39 (2.,06)	9.,94 (2.,86)	10.,41 (3.,14)	0.088	0.423 (df = 2,73)
ST	8.,45 (3.,13)	8.,91 (3.,47)	9.,7 (3.,06)	0.773 (df = 1,30)^d^	0.773 (df = 1,30)^d^
*Recognition (n = 39*, *37)*					
CR	12.,65 (2.,29)	12.,12 (2,.80)	12.,53 (2.,38)	0.353	0.925
ST	12.,59 (2.,18)	13.,09 (1.,88)	12.,55 (2.,09)	0.228 (df = 1,33)	0.296 (df = 2,72)
**TMT-B** (n = 37, 37)					
CR	69,.19 (26.,89)	66.,69 (29.,51)	66.,53 (24.,19)	0.070	0.105
ST	66.,10 (20.,57)	58.,86 (18.,14)	55.,75 (16.,86)	0.060 (df = 1,31)	0.568 (df = 2,70)
**FAST** (n = 38, 36)					
CR	26.,47 (8.,48)	23.,41 (7.,68)	23.,5 (9.,83)	0.588	0.009*
ST	28.,71 (12)	22.,48 (12.,21)	23.,55 (13.,54)	0.424 (df = 1,34)	0.510 (df = 2,69)
**RVP**					
*Accuracy (B') (n = 35*, *37)*					
CR	0.,96 (0.,06)	0.,93 (0.,10)	0.,83 (0.,48)	0.972	0.128
ST	0.,95 (0.,05)	0.,95 (0,.06)	0.,85 (0.,44)	0.178 (df = 1,29)	1.000 (df = 2,61)
*Time to correct response (ms) (n = 36*, *37)*					
CR	403.,95 (65.,13)	384.,63 (54.,21)	393.,57 (76.,62)	0.716	0.821
ST	423.,82 (79,.10)	412.,83 (86.,3)	410.,23 (100.,23)	0.454 (df = 1,30)	0.973 (df = 2,68)
*Sensitivity (A') (n = 37*, *37)*					
CR	0,.92 (0.,03)	0.,93 (0.,05)	0.,92 (0.,05)	0.081	0.373
ST	0.,90 (0.,06)	0.,86 (0.,21)	0.,93 (0.,06)	0.263 (df = 1,31)	0.253 (df = 2,73)

Abbreviations: SD, standard deviation; CR, cognitive remediation; ST, standard treatment; RAVLT, Rey Auditory Verbal Learning Test; TMT-B, Trail Making Test part B; FAST, Functioning Assessment Short Test; RVP: Rapid Visual Information Processing.

^a^Degrees of freedom (df) are identical for time and time by group unless otherwise specified.

^b^p values based on ANCOVA analyses.

^c^p values based on mixed models analyses.

^d^Demonstrates difference between groups at week 12 or 26 adjusted for significant baseline differences rather than interaction between time and group.

n values may differ between tests and time of measurement according to attendance or fatigue. The two n values given for each test reflects the number of participants analysed at week 12 and 26, respectively.

*Significant p values.

### Secondary outcomes

Baseline performance on RVP, TMT-B and FAST showed no differences between groups (p-values>0.478). There was a trend towards improvement in TMT-B performance in the ST group compared with CR from baseline to week 12 (F(1,31) = 3.82, p = 0.060), which disappeared at week 26 (p = 0.568). FAST and RVP showed no effects of CR over ST from baseline to week 12 (p-values>0.178) or week 26 (p-values>0.253).

### Tertiary outcomes

Tables 3 and 4 displays results for tertiary outcomes. Baseline performance on tertiary outcomes was comparable across the two groups (p-values>0.124). CR enhanced subjective sharpness/mental acuity on the CPFQ compared with ST from baseline to week 12 (F(1,30) = 7.017, p = 0.013; partial Eta squared = 0.189) and this effect was maintained at follow-up (F(2,63) = 3.775, p = 0.028). There was a trend towards improvement in verbal fluency letter ‘S’ in the CR vs. ST groups from baseline to week 12 (F(1,30) = 3.788, p = 0.061), which became significant at follow-up (F(2,67) = 5.683, p = 0.005; β = 4.47, 95% CI 1.82–7.13, p = 0.001). CR also improved the psychological domain of the WHOQOL-Bref (F(2,67) = 3.172, p = 0.048; β = 1.79, 95% CI 0.14–3.44, p = 0.034) at follow-up, and there was a strong trend towards improved self-reported stress as measured with PSS (F(2,67) = 2.988, p = 0.057) at follow-up. There were no differences between groups on additional measures of cognitive and psychosocial function or quality of life from baseline to week 12 (p-values>0.203) or week 26 (p-values>0.102).

**Table 3:** Between-group differences statistics for measures of objective cognitive function (tertiary outcomes).

				Time (weeks 0–12)	Time (weeks 0–26)
	Week 0 (baseline)	Week 12	Week 26	Time—by group^b^	Time—by group^c^
	Mean (SD)	Mean (SD)	Mean (SD)	p value^a^	p value^a^
**Objective cognitive function**					
**TMT-A** (n = 37, 37)					
CR	35.25 (13.44)	31.56 (10.79)	29.88 (12.2)	0.812	0.004*
ST	30.43 (7.28)	28.05 (4.82)	25.55 (5,58)	0.740 (df = 1,31)	0.836 (df = 2,70)
**RBANS digit span** (n = 37, 37)					
CR	10.19 (1.72)	10 (1.86)	9.71 (2.23)	0.997	0.220
ST	9.52 (2.23)	9.81 (1.99)	9.15 (1.73)	0.452 (df = 1,31)	0.747 (df = 2,70)
**RBANS coding** (n = 37, 37)					
CR	50.56 (11.03)	51.63 (11.72)	50.94 (12.47)	0.055	0.013*
ST	47.57 (10.13)	49.24 (9.31)	51 (9.56)	0.888 (df = 1,31)	0.940 (df = 2,68)
**WAIS-III Letter-Number Sequencing** (n = 37, 37)					
CR	10.75 (2.52)	10.88 (2.55)	10.71 (2.59)	0.151	0.321 (df = 2,69)
ST	10.43 (2.64)	10.71 (2.61)	11.25 (2.85)	0.692 (df = 1,31)	0.369 (df = 2,68)
**Verbal Fluency** (n = 36, 37)					
*Letter S*					
CR	13.5 (3.8)	15.13 (4.84)	16.94 (4.88)	0.503	0.144 (df = 2,68)
ST	16.75 (6.1)	15.85 (5.42)	15.45 (6.53)	0.061 (df = 1,30)	**0.005** * (df = 2,67)
*Letter D*					
CR	10.44 (3.58)	10.88 (3.63)	10.71 (3.44)	0.876	0.465
ST	10.9 (5.74)	11.45 (5.23)	11.3 (5.59)	0.923 (df = 1,30)	0.877 (df = 2,68)
*Animals*					
CR	20.5 (4.73)	21 (7.78)	22.24 (6.44)	0.856	0.565
ST	22.2 (5.45)	23.05 (6.73)	22.05 (6.26)	0.869 (df = 1,30)	0.500 (df = 2,69)
*Streets*					
CR	17.75 (5.73)	18.75 (5.56)	20.82 (5.7)	0.060	0.064
ST	17.75 (6.85)	17.35 (5.93)	18.05 (6.4)	0.433 (df = 1,30)	0.606 (df = 2,69)
**FERT** (n = 35, 37)					
*Accuracy*, *all emotions*					
CR	20.86 (3.11)	20.36 (5.58)	21.16 (3.35)	0.857	0.387
ST	19.54 (4.16)	20.78 (2.45)	20.76 (2.75)	0.233 (df = 1,29)	0.406 (df = 2,68)
*Mean reaction time*, *all emotions*					
CR	1366.47 (350.94)	1401.97 (432.65)	1386.46 (445.99)	0.553	0.084
ST	1399.82 (245.65)	1313.68 (296.1)	1263.29 (239.9)	0.081 (df = 1,29)	0.213 (df = 2,68)
**SRT** (n = 36, 37)					
*Accuracy*					
CR	98.5 (1.37)	98.81 (1.87)	98.76 (1.82)	0.636	0.432 (df = 2,68)
ST	98.05 (2.31)	986 (1.39)	98.7 (1.45)	0.526 (df = 1,30)	0.952 (df = 2,67)
*Mean reaction time*					
CR	265.02 (61.71)	263.91 (40.17)	279.16 (56.85)	0.659	0.240
ST	249.3 (39.23)	267.01 (53.01)	267.58 (54.79)	0.331 (df = 1,30)	0.606 (df = 2,68)
**DMS** (n = 36, 37)					
*Accuracy (percent correct)*					
CR	89.06 (9.17)	93.13 (6.02)	93.82 (4.16)	0.739	0.082
ST	88.25 (9.07)	89.5 (7.05)	90 (8.43)	0.379 (df = 1,30)	0.179 (df = 2,68)
*Mean reaction time (mean correct latency)*					
CR	2748.73 (704.2)	3018.19 (915.24)	3079.77 (669.08)	0.998	0.683
ST	3186.88 (793.28)	3016.94 (823.43)	2980.52 (1007.77)	0.203 (df = 1,30)	0.139 (df = 2,68)
**SWM** (n = 36, 37)					
*Mean score*, *'between errors' 4–8 boxes*					
CR	7.77 (5.95)	6.25 (6.72)	4.65 (4.51)	0.901	0.011*
ST	6.57 (6.59)	4.95 (4.76)	5.3 (6.05)	0.976 (df = 1,30)	0.415 (df = 2,68)
*Strategy*					
CR	31.19 (6.66)	28.38 (7.62)	27.71 (6.18)	0.805	0.012*
ST	30.05 (7.05)	28.6 (6.52)	29.15 (7.68)	0.487 (df = 1,30)	0.496 (df = 2,68)

Abbreviations: SD, standard deviation; CR, cognitive remediation; ST, standard treatment; TMT-A, Trail Making Test part A; RBANS, Repeatable Battery for the Assessment of Neuropsychological Status; WAIS, Wechsler Adult Intelligence Scale; FERT, Facial Expression Recognition Task; SRT, Simple Reaction Time; DMS, Delayed Matching to Sample; SWM, Spatial Working Memory.

^a^Degrees of freedom (df) are identical for time and time by group unless otherwise specified.

^b^p-values based on ANCOVA analyses.

^c^p-values based on mixed models analyses.

n-values may differ between tests and time of measurement according to attendance or fatigue. The two n-values given for each test reflects the number of participants analysed at week 12 and 26, respectively.

*Significant p-values.

**Table 4:** Between-group differences statistics for self-reported cognitive function, quality of life and psychosocial function (tertiary outcomes).

				Time (weeks 0–12)	Time (weeks 0–26)
	Week 0 (baseline)	Week 12	Week 26	Time—by group^b^	Time—by group^c^
	Mean (SD)	Mean (SD)	Mean (SD)	p value^a^	p value^a^
**Subjective cognitive function**					
**CPFQ** (n = 36, 34)					
*Total scores*					
CR	26.41 (4.33)	19.88 (3.62)	23.07 (5.6)	0.195	<0.001*
ST	26.21 (4)	21.63 (5.33)	23.63 (4.89)	0.286 (df = 1,32)	0.408 (df = 2,67)
*Sharpness/mental acuity*					
CR	3.76 (0.75)	3.12 (0.7)	3.13 (0.92)	0.541	0.029*
ST	3.42 (0.9)	3.58 (1.12)	3.32 (0.89)	**0.010** * (df = 1,32)	**0.032** * (df = 2,67)
**CFQ** (n = 35, 35)					
CR	53.13 (12.88)	51.06 (13.2)	46.93 (13.67)	0.342	0.003*
ST	58.05 (15.06)	57.21 (15.88)	53.45 (15.1)	0.595 (df = 1,31)	0.968 (df = 2,66)
**Quality of Life**					
**WHOQOL-Bref** (n = 35, 35)					
*Total scores*					
CR	83.88 (12.61)	83.13 (14.2)	91.33 (12.77)	0.686	0.014*
ST	84.21 (11.01)	81.53 (17.85)	84.95 (14.11)	0.644 (df = 1,31)	0.323 (df = 2,67)
*Psychological domain*					
CR	11.71 (2.79)	11.33 (3.28)	13.47 (3.42)	0.920	0.038*
ST	11.33 (2.44)	10.91 (3.81)	11.1 (2.61)	0.967 (df = 1,31)	**0.048** * (df = 2,67)
**EQ-5D** (n = 36, 35)					
*Total scores*					
CR	6.5 (1.27)	6.75 (1.29)	6.47 (1.25)	0.231	0.343
ST	7 (1.26)	7.45 (1.93)	7.6 (1.79)	0.749 (df = 1,32)	0.636 (df = 2,68)
*VAS score*					
CR	73.38 (21.39)	57.25 (30.4)	63.47 (26.88)	0.257	0.012*
ST	72.65 (21.66)	63.6 (23.57)	75.45 (18)	0.483 (df = 1,32)	0.384 (df = 2,70)
**PSS** (n = 35, 35)					
CR	19.31 (5.64)	19 (6.61)	14.73 (6.2)	0.859	0.132
ST	21.89 (8.07)	22.63 (9.71)	22.9 (8.08)	0.683 (df = 1,31)	0.057 (df = 2,67)
**WSAS** (n = 35, 35)					
CR	19.69 (6.76)	20.75 (6.91)	17.93 (7.22)	0.791	0.372
ST	21 (6.21)	21.53 (8.67)	20.6 (10.88)	0.817 (df = 1,31)	0.689 (df = 2,67)

Abbreviations: SD, standard deviation; CR, cognitive remediation; ST, standard treatment; CPFQ, Massachussetts General Hospital Cognitive and Physical Functioning Questionnaire; CFQ, Cognitive Failures Questionnaire; WHOQOL-Bref, World Health Organization Quality of Life BREF; EQ-5D-3L, European Quality of Life—5 Dimensions—3 Levels; PSS, Cohen's Perceived Stress Scale; WSAS, Work and Social Adjustment Scale.

^a^Degrees of freedom (df) are identical for time and time by group unless otherwise specified.

^b^p-values based on ANCOVA analyses.

^c^p-values based on mixed models analyses.

n-values may differ between tests and time of measurement according to attendance or fatigue. The two n-values given for each test reflects the number of participants analysed at week 12 and 26, respectively.

*Significant p-values.

### Explorative sub-group analyses

Following the negative findings for the a priori defined analyses, a series of exploratory sub-group analyses of verbal memory performance from baseline to week 12 were conducted. These analyses revealed no differential effects of CR in participants with BD type I versus type II (p-values>0.596); in participants who had continued their medication without any alterations throughout the trial (n = 37) (p-values>0.314); when excluding participants using antipsychotics or benzodiazepines from the analysis (CR, n = 8; ST, n = 10) (p-values>0.719); when including only CR participants who had attended a minimum of 10 sessions (>80% attendance) in the analyses (n = 11) (p-values>0.375).; or in a subgroup of participants in full remission through to week 12 (n = 24) (HDRS-17 and YMRS scores of <7) (p-values>0.381).

## Discussion

The present study is the first to investigate the effects of CR on cognitive dysfunction in BD in a randomised, controlled design. In contrast with our hypothesis, the present trial revealed no effects of CR vs. ST on verbal memory (primary outcome), sustained attention, executive function or psychosocial function (secondary outcomes). CR was, however, associated with long-term improvement of subjective sharpness/mental acuity, verbal fluency performance and one aspect of quality of life (tertiary outcomes). Additional explorative sub-group analyses also revealed no effects of CR vs. ST on the primary study outcome in participants with BD type I vs. type II, participants on stabile medication or with no use of benzodiazepines or antipsychotics, in those who were fully remitted or in CR participants with >80% group attendance.

Our negative results corroborate the demonstration that 21 weeks of FR over ST had no effect on any measures of cognitive function [16]. Although three preliminary studies of the effects of CR in affective disorders [17–19] pointed to some cognitive benefits, there is no convincing evidence that group-based, short-term CR (or FR) can remediate cognitive dysfunction in BD. In contrast, cognitive improvement has been shown with CR in patients with schizophrenia who exhibit more severe cognitive dysfunction than patients with BD [53,54]. It is possible that our CR format was not long or intensive enough given the longer-term (average 16.7 weeks), more frequent sessions (2.2 per week) and mainly individualized CR studies in schizophrenia [15]. We would suggest that future trials employ sufficient screening methods to ensure inclusion of participants who demonstrate objective cognitive deficits in addition to subjective cognitive difficulties as they are more likely to benefit from a CR intervention. Furthermore, future trials should investigate if CR delivered in a more intensive and individualised format with supervision of homework would demonstrate more beneficial cognitive effects in individuals with BD than suggested in the present study since our study showed that short-term group-based CR is most likely ineffective.

Despite the overall negative outcome of the trial, participants in the CR group did show improved subjective sharpness/mental acuity, verbal fluency and psychological quality of life and there was a strong trend toward decrease in self-reported stress in this group compared with ST. The intervention must, therefore, still be considered ethical and could potentially improve some aspects of subjective cognitive and psychosocial function.

### Limitations

The primary limitation of the study was the relatively small sample size (n = 40) which turned out to provide suboptimal statistical power for the primary outcome analysis. We had based the original sample size calculation before trial start on the assumption that the standard deviation of the change in RAVLT total scores would be 4 points. However, post-hoc analysis after trial completion showed that the standard deviation of the RAVLT change scores was greater than expected (approximately 8 points), and that the statistical power to detect a clinically relevant change in RAVLT was therefore suboptimal. Therefore, to assess how confidently we can conclude that CR produced no positive change in verbal memory relative to ST, we calculated the 95% confidence interval for the change in RAVLT total score in the CR versus ST groups from baseline to week 12. While the CR group showed a change in RAVLT total recall of -0.55 points, the ST group should an increase in these scores of 2.49 scores (see Table 2). The difference between the groups in the change in RAVLT total scores was thus -3.19 points, with a standard deviation of 7.9 points of the change scores across the entire sample. Based on this, the 95% confidence interval was calculated to be -5.69 to -0.70. This shows that we can rule out with 95% certainty that the present short-term CR intervention would have produced any greater change in RAVLT total scores than -0.70 (i.e. no effect) even with optimal statistical power. It is therefore highly unlikely that an increase in sample size could have rendered any beneficial (or even clinically relevant) effects of the CR on verbal memory. Another limitation is that we used only subjectively reported cognitive dysfunction as an inclusion criterion and did not assess whether these were accompanied by objective cognitive impairment at baseline. Indeed, we found after trial start that there was no correlation between subjective and objective measures of cognitive dysfunction [14,55], suggesting that it is not always the individuals who report the worst cognitive symptoms who are most objectively impaired and vice versa. The rationale for choosing subjective cognitive dysfunction as an inclusion criterion was (i) that only patients who experience cognitive difficulties would be motivated for this treatment and (ii) there is no existing consensus of inclusion criteria in randomised controlled trials targeting cognition. Post-hoc comparisons of baseline cognitive performance with international normative data showed that our participants, despite their cognitive complaints, had no objective cognitive dysfunction in executive function or sustained attention as reflected by TMT-B and RVP performance within the normal range (of age-matched individuals (norm material from Jørgensen [56] and Cambridge Cognition). Nevertheless, verbal memory was significantly impaired relative to meta-norms (about 1 SD under the mean of healthy gender and age-matched individuals [57]) but still showed no beneficial effects of CR vs. ST. More specifically, a subgroup of 14 participants (35%) showed impairment (RAVLT>1 SD under the meta-norm) while the remaining 26 patients (65%) displayed normal function (meta-norms from [57]. Post-hoc sub-group ANCOVA analyses adjusted for stratification variables and mood excluding ‘high performers’ (>0.5 SD better than norms) still revealed no significant effects of CR vs. ST on these measures of attention, executive and memory function (p-values>0.130). Accordingly, a recent study found that individuals with BD can be divided into three clusters according to level of cognitive dysfunction: a globally impaired group (global) (39.7%) with severe and diffuse cognitive dysfunction, a selectively impaired group (selective) (31.6%) with modest deficits on specific cognitive domains and an intact group (intact) (28.7%) with comparable performance to healthy controls on all domains [58]. In our study, a comparable group showed selective impairment whereas more of our participants displayed intact cognitive function compared with the cohort of BD individuals in the study by Burdick and colleagues. Based on this and the emerging evidence for poor correlation between subjective and objective dysfunction from the present and other trials, we suggest that future trials should implement an additional brief, objective cognitive screening tool for correct identification of participants with objective cognitive impairment. It is conceivable that the absence of objective deficits in a large proportion of our participants in comparison with normative data could have reduced the scope for cognitive improvement with CR vs. ST because of ceiling effects. Indeed, neuropsychological impairment in BD at baseline has been shown to predict cognitive improvement in response to CR treatment [17]. However, it is unlikely that such ceiling effects can explain the negative findings of the present trial; sub-group analyses excluding the ‘intact’ groups (including n = 31, n = 10 and n = 15 in analyses of RAVLT, RVP-B and TMT-B, respectively) showed no effect of CR vs. ST, and the impaired CR groups actually displayed a slight reduction in verbal memory and sustained attention from baseline to post-treatment. Taken together, this pattern of results suggests that the negative outcome represented a true absence of effects of our CR treatment rather than type 2 errors due to our relatively small sample size.

Furthermore, it is possible that longer duration and more intensive treatment may have rendered a positive signal. However, we originally chose a short-term treatment assuming that the more subtle cognitive deficits in BD compared with schizophrenia would require shorter and perhaps less intensive treatment.”

Finally, participants were included in the trial even if they were only in partial remission. Albeit we did adjust for changes in HDRS-17 and YMRS in the analyses it might have influenced the present negative findings e.g. affected motivation. Indeed, Burdick et al. [12] found no overall effects of pramipexole on cognitive function in a group of both fully and partially remitted participants; however, there was a significant treatment effect in a sub-group of euthymic participants, suggesting that remission facilitates cognitive improvement in response to pharmacological treatment. Nevertheless, we recently found robust cognitive improvement across several cognitive domains in response to EPO vs. saline treatment in a group of only partially remitted patients with BD [14], suggesting that full remission is not necessary for cognitive benefits of an intervention per se.

## Conclusions

Short-term group-based CR delivered at a weekly basis to fully or partially remitted individuals with BD with subjective cognitive complaints did not improve cognitive function in comparison with ST. Despite participants’ self-reported cognitive difficulties they showed no objective cognitive dysfunction in comparison with healthy, age-matched norm groups; nevertheless post-hoc assessment of treatment effects in the group who showed selective and global cognitive impairment still revealed no effects of CR over ST. This suggests that it was characteristics of the implemented CR treatment (group-based format, short-term) that accounted for the negative findings.

Based on these findings, further studies are warranted to investigate the effects of longer-term, individualised CR treatments. Such studies should also include a short screening instrument to correctly identify patients with objective cognitive impairment who may have most benefit of the treatment.

## Supporting Information

S1 CONSORT Checklist (DOC)

S1 Protocol (DOC)
