# Supplementary material for: Effects of Short-Term Cognitive Remediation on Cognitive Dysfunction in Partially or Fully Remitted Individuals with Bipolar Disorder: Results of a Randomised Controlled Trial
Source: PLoS One. 2015 Jun 12;10(6):e0127955. doi: 10.1371/journal.pone.0127955 (PMC4467086; doi:10.1371/journal.pone.0127955)
Supplement: S1 Protocol — (DOC) [file pone.0127955.s002.doc]

Effects of cognitive remediation on cognitive dysfunction in partially or fully remitted patients with bipolar disorder: study protocol for a randomised controlled trial.

**Kirsa M. Demant, MSc**

**Glennie Marie Almer, MSc**

**Maj Vinberg, MD, PhD**

**Lars Vedel Kessing, professor, MD, DMSc**

**Kamilla W. Miskowiak, MSc, PhD**

Copenhagen Affective Disorder Clinic, Psychiatric Centre Copenhagen, Copenhagen University Hospital, Rigshospitalet, Copenhagen, Denmark.

Corresponding author:

Kirsa M. Demant, Psychiatric Centre Copenhagen, Copenhagen University Hospital, Rigshospitalet, Copenhagen, Denmark. Email: kirsa.moerkeberg.demant@regionh.dk

Email addresses:

KD: kirsa.moerkeberg.demant@regionh.dk

GMA: glennie.marie.almer@regionh.dk

MV: maj.vinberg@regionh.dk

LVK: lars.vedel.kessing@regionh.dk

KM: kamilla.miskowiak@regionh.dk

## Abstract

## Background

A large proportion of patients with bipolar disorder suffer from persistent cognitive dysfunction, such as memory, attention and planning difficulties even during periods of full remission. The aim of the present trial is to investigate whether cognitive remediation, a new psychological treatment, improves cognitive function and, in turn, psychosocial function in patients with bipolar disorder in partial or full remission.

## Methods

The trial has an evaluator-blind, randomised, between-groups design. Forty patients with bipolar disorder in full or partial remission, age 18-50 years, who report having moderate to severe cognitive difficulties, are recruited. Patients are randomised to receive weekly group-based cognitive remediation treatment over twelve weeks in addition to standard treatment or standard treatment alone. Both groups undergo neurocognitive testing and functional magnetic resonance imaging (fMRI) at baseline, post-treatment (week 12) and at follow-up (week 26). The primary outcome is improved verbal memory measured with the Rey Auditory Verbal Learning Test (RAVLT) from baseline to post-treatment. With inclusion of 40 patients we obtain 86% power to detect a clinically relevant difference in verbal memory between groups. The secondary outcomes are improved attention, executive function and psychosocial function measured with Rapid Visual Information Processing (RVP), Trail Making Test (TMT) part B and the Functional Assessment Short Test (FAST), respectively. Tertiary outcomes are improvement on additional neuropsychological tests of memory, attention, executive function and facial expression recognition as well as questionnaires measuring subjective cognitive difficulties, stress, coping strategies, personality traits, depressive symptoms and quality of life.

**Discussion:**

This is the first randomised controlled trial to evaluate the effects of cognitive remediation on cognitive function in patients with bipolar disorder who despite being in full or partial remission experience persistent cognitive difficulties.

# **Keywords**

Cognitive remediation, computer assisted cognitive remediation, cognitive training, bipolar disorder, affective disorders, cognitive function, cognitive dysfunction, cognitive difficulties, psychosocial function, occupational function.

## Background

Worldwide, Bipolar Disorder (BD) is among the ten leading causes of reduced functional ability (World Health Organisation (WHO) [1]. Despite medical treatment with mood stabilisers, newer antipsychotics and antidepressants, a large proportion of patients with BD experience persistent and debilitating cognitive dysfunction [2-4]. In particular, trait-related deficits have been shown in verbal memory, sustained attention and executive function [5;6] as well as in social cognition [7;8]. These cognitive deficits may not simply be side effects of the medical treatment but may also represent a core feature of the psychopathology [9;10]. It has been estimated that 30-60% of bipolar patients have trait-related cognitive deficits and that these reduce their occupational and social functioning [11-13] and quality of life [14]. There is an urgent clinical need for new treatment strategies that target cognitive enhancement in BD.

Cognitive remediation (CR) is a new psychological treatment which aims to improve cognitive function, compensational and coping skills and, consequently, psychosocial function. CR has been studied extensively in patients with schizophrenia; a recent meta-analysis of 40 studies demonstrated that CR leads to lasting improvement of cognitive and social function levels in this patient group (effect size [ES]= 0.45; 95% confidence interval [CI]=0.31-0.59) [15]. Another meta-analysis of 16 studies of CR in mixed groups of patients with affective disorders, affective psychosis, schizophrenia and/or schizo-affective disorders suggests that patients with BD can achieve beneficial effects of CR that are equivalent to those observed in patients with schizophrenia (ES= 0.32; 95% CI=0.20-0.43) [16]. Four of these studies included patients with affective disorders; of these one study in unipolar disorder showed that memory improvement following CR was associated with increased occupational function [17]. Similarly, improved psychosocial and occupational functioning was found in a small, uncontrolled trial of 18 patients with BD [18] and in a small, controlled trial with a mixed sample of patients with unipolar depression and BD [19] with large effect size; (partial eta squared=0.45). Nevertheless, the studies included in the meta-analysis provide only preliminary evidence.

Recently, a multi-centre RCT investigated the effects of functional remediation versus either psychoeducation or standard care on psychosocial function in euthymic patients with BD [20]. Although patients in the functional remediation group showed functional improvement compared with those in standard care (with small effect size; d’=0.3), this was not significantly different from the improvement observed in the psychoeducation group. In addition, assessment of cognitive function revealed no beneficial effects of functional remediation over psychoeducation or standard care [20].

The aim of the present trial is therefore to investigate if CR has beneficial effects on cognitive and psychosocial function in patients with fully or partially remitted BD who experience persistent cognitive difficulties. The rationale for inclusion of this remitted patient group is that their trait-related cognitive deficits are not successfully targeted by current treatments, as opposed to the state-related deficits in acute affective episodes, which may in some cases resolve fully with affective symptom reduction [2-4]. Beneficial effects on trait-related cognitive deficits would hence point to future implementation of CR for remitted patients with BD in order to facilitate these patients’ cognitive abilities and capacity to cope with education, work and everyday functioning.

## Hypotheses

The hypotheses of the present study are that CR, in comparison with standard treatment, will:

1. Enhance verbal memory (primary outcome) measured with the Rey Auditory Verbal Learning Test (RAVLT) from baseline to week 12
2. Improve sustained attention, executive function and psychosocial function (secondary outcomes) measured with the Rapid Visual Information Processing (RVP), Part B of the Trail Making Test (TMT) and Functional Assessment Short Test (FAST)
3. Improve additional measures of attention, executive function and facial expression recognition measured with a comprehensive neurocognitive test battery; and improve self-reported cognitive and psychosocial function measured with questionnaires

##### *Biomarkers of treatment effects*

In addition, we wish to investigate the below biomarkers of potential beneficial effects of CR on the above outcome parameters: plasma Brain Derived Neurotrophic Factor (BDNF), stress response as reflected by morning awakening cortisol and the neuroanatomical underpinnings of memory, attention, executive function and emotional processing. We hypothesise that potential beneficial effects of CR are associated with (A) increase in plasma BDNF, (B) reduction in morning awakening cortisol and/or (C) changes in the neuroanatomical underpinnings of memory, attention, executive function and emotional processing (see details under section ’Brain biomarkers of potential treatment effects’).

## Methods

### Study design

Patients will be randomised (1:1) to receive either CR in a group setting or standard treatment in an evaluator-blind between-groups design.

*Participants and screening*

Patients will be recruited mainly through the Copenhagen Affective Disorder Clinic, Psychiatric Centre Copenhagen, Copenhagen University Hospital, Rigshospitalet, Copenhagen, Denmark. The centre receives patients with complex affective disorders from the whole Capital Region. Patients will receive written information about the project and will be given the opportunity to ask questions before deciding on participation in the project. Further information is available during the entire course of investigation from any of the researchers involved in the project. Patients will be screened with a medical check and a psychiatric interview using Schedules for Clinical Assessment in Neuropsychiatry [21] to confirm diagnosis, and the Hamilton Depression Rating Scale, 17 items (HDRS-17) [22] and the Young Mania Rating Scale (YMRS) [23] to determine the severity of depressive and manic symptoms.

We will recruit a minimum of 44 patients with the assumption of a 10% dropout rate, leaving us with 40 completers. We include patients with BD (as defined by ICD-10 diagnostic criteria), age 18-50 years, who are in full or partial remission (defined as HDRS-17 and YMRS scores of <14) and have subjective complaints of cognitive difficulties of moderate to severe degree (score of >4) in at least 2 of 7 cognitive domains on the Massachusetts General Hospital Cognitive and Physical Functioning Questionnaire (CPFQ) [24]. Exclusion criteria are: current ECT-treatment, current substance or alcohol abuse, schizophrenia, schizoaffective disorder or significant suicide risk. Patients are permitted to take antidepressant, lithium, antipsychotic medication and benzodiazepines corresponding to < 22.5 mg Oxazepam daily.

**Interventions**

### Cognitive remediation treatment

Patients randomised to the CR group will get CR in addition to standard treatment. A group of clinical psychologists at the Copenhagen Affective Disorder Clinic manualised the CR treatment. The cognitive remediation programme is based on a treatment programme for patients with schizophrenia [25], experience with cognitive rehabilitation in brain damage and thorough clinical and research-based knowledge of cognitive difficulties in bipolar disorder. Before study initiation we conducted a small pilot study including six patients between September and December 2009 with the purpose of testing and revising the treatment manual and session structure and to define treatment length. CR was then designed to consist of twelve weekly group sessions of two hours each, followed by a booster session four weeks after treatment completion. Groups include between five and eight patients and are run by two group therapists; a specialist in psychiatry with more than 20 years of clinical expertise with bipolar patients and a clinical psychologist trained in neuropsychology.

The programme have three main components: 1) psychoeducation and awareness of cognitive dysfunction in bipolar disorder, including an emphasis on discovering each patient’s individual profile of cognitive dysfunction; 2) training of compensatory and adaptive strategies in relation to cognitive dysfunction; and 3) computer-assisted cognitive training using RehaCom [26;27]. The computerised exercises chosen for our intervention target attention and concentration, memory and learning and executive function. Most exercises are highly ecologically valid as they focus on everyday life situations, thereby optimising the translation from computer training to real life cognitive demands. For example planning activities, shopping groceries, counting, memorising short articles, memorising people’s faces and their names, job titles and phone numbers etc. The level of difficulty is automatically customised to each patient and increases as the patients’ performance improves. Furthermore, employing computer exercises as part of the treatment provides easily accessible homework and enables an individually tailored training despite the group-based intervention.

A number of general principles for the CR sessions were established and are followed by the group therapists to facilitate learning: No use of power point slides while the therapists give brief lectures on a given topic in order to minimize the strain on the patient’s divided attention. To account for the patient’s limited attention span, the therapists are allowed to talk for a maximum of 5-10 minutes without patient involvement, after which patients are required to engage actively in the session. Each session consists of 120 minutes including a 15 minutes break in-between. Homework, which practises attention, memory and executive function consist of computer training, mindfulness exercises and reading a textbook about cognitive dysfunction in bipolar disorder. Occasionally, the use of new compensatory and adaptive strategies are also included as part of the homework between sessions. For homework assignments it is discussed and planned in each session how the patient can most likely succeed in fulfilling the assignments at home. This includes planning the time of day and for how long each patient will read and train the assigned computer tasks. For the homework reading tasks, each section of the book is assigned as homework twice in order to facilitate the application of adaptive and compensatory strategies as well as to enhance the possibility of learning and remembering the contents.

The twelve sessions are divided into four main topics. The first two sessions consist of an introduction to cognitive function and dysfunction and to the principles of cognitive remediation. In session three to five, attention and concentration are the main focus. An emphasis is placed on the importance of attention as a foundation for other areas of cognitive function such as memory. The main elements in these sessions are psychoeducation on the variation in attentional function in bipolar disorder, basic neuropsychological knowledge concerning various aspects of attentional function, computer training and compensatory and adaptive strategies. Examples of such strategies for enhancing attentional function are reading with and without background music in order to discover what works best, allowing frequent breaks during attention straining tasks such as setting the alarm when reading, and limiting oneself to carry out one task at a time rather than attempt to multitask a number of activities or initiate activities that are not completed. Finally, therapists train patients in mindfulness meditation as a mean to enhance attentional capacity [28], and in the subsequent sessions, a brief mindfulness meditation was carried out at the beginning of each session.

Sessions six to eight address memory and learning. During these sessions, various exercises on remembering visual and auditory verbal input are carried out. These exercises include a reading task where patients are handed out a brief newspaper article on a relevant topic ten minutes before the fifteen minutes break during a session. The timing is to ensure a small time gap between encoding and recall of the content of the article. Patients are asked to read the article with the intention of giving a brief resume in front of the rest of the group after the break. Before commencing the task we refresh memory techniques such as reading aloud, taking brief notes, using visual imagery in order to remember key points etc. Another exercise is a conversational task, also carried out just before the fifteen minutes break during sessions. In this exercise, patients work in pairs; one of them pretending to be a close friend or relative and the other asking questions to get to know this person. Again, following the fifteen minutes break they are asked to tell the rest of the group about the person they had "interviewed". The idea of pretending to be another person is to ensure that the listening patient does not have beforehand knowledge of the conversation partner.

Brief talks are given on the various aspects of memory such as long term versus short-term memory and working memory, and verbal versus visuo-spatial memory. Introduction to establishing structure in everyday life is followed by homework in this area. Homework includes establishing a routine of using only one calendar, whether it is an electronic version or a traditional paper calendar, rather than using multiple calendars, which may cause confusion. Furthermore, patients are instructed in efficient use of a calendar by including all appointments plus time for rest and physical exercise, grocery shopping etc. Possible cognitive side effects of medication and substance abuse are discussed.

Session nine to twelve are dedicated to executive function in everyday life. Thorough discussions of where executive dysfunction arises provide the foundation for individualised homework assignments in order to improve these areas of functioning. In session, we carry out troubleshooting as a group-based exercise. One patient mentions a problem such as being incapable of cooking a meal or always being late for appointments feeling stressed out. With thorough guidance from the group therapists we break down the problem into as many subcomponents as possible and try to conjure up new and creative solutions to try out as homework in between sessions. The homework assignments are followed up in great detail. Also, at this point in the treatment programme, reading of fiction as cognitive training of attention and verbal memory is introduced. Each patient is encouraged to choose a book as a homework assignment and a detailed plan for the reading process is established. An emphasis is placed on making the treatment programme as individualised as possible.

### Standard treatment group

Patients in the control group receive standard treatment consisting of the standard out-patient mental health services routines in The Capital Region of Denmark either at the Copenhagen Affective Disorder Clinic, community psychiatric centres or private specialists in psychiatry. Standard treatment involves psychopharmacological mood stabilization and relapse prevention and, in our clinic and most community psychiatric centers, psychoeducation. In contrast to the intervention group, standard treatment does not involve any specific cognitive training.

## Randomisation

Pharma Consulting Group [29] performed the randomisation of the patients with stratification for age (<35 years) and years of education (<15 years).

## Blinding

Study personnel involved in the evaluation of outcomes are blinded to treatment allocation. Blinding is maintained throughout the study, data management, outcome assessment and data analysis.

**Outcome assessments**

The main study outcomes (cognitive and psychosocial function) and biomarkers of potential treatment effects (plasma BDNF, cortisol levels and neuroanatomical underpinnings) will be measured at baseline, after completion of treatment (week 12) and at follow-up (week 26). We also rate affective symptoms at these three time points and additionally at weeks 6 and 20 to uncover potential mood swings in the periods between outcome assessments

### Neurocognitive function

Patients undergo neurocognitive testing with a comprehensive test battery including the following RAVLT subtests: total recall across the five learning trials (I-V), RAVLT recall following interference (trial VI), recall following a 30 minute delay and recognition from baseline to week 12 [30;31]; TMT parts A and B [32]; Repeatable Battery of the Assessment of Neuropsychological Status (RBANS) digit span and coding [33]; WAIS-III Letter-Number Sequencing [34]; Verbal and Semantic Fluency Tests [35]; Danish Adult Reading Test (DART) [36]; the computerised test Facial Expression Recognition Task (FERT) from the Oxford Emotional Test Battery (P1Vital; Oxford); and the following computerised tests from Cambridge Cognition (CANTAB): Simple Reaction Time (SRT); RVP; Delayed Match-to-Sample (DMS) and Spatial Working Memory (SWM).

### Psychosocial function

Psychosocial function will be measured with FAST [37], which is specifically developed and validated for bipolar disorder.

### Questionnaires

Self-reported cognitive difficulties, stress, coping strategies, personality traits, depressive symptoms and quality of life are measured using the following questionnaires: CPFQ; State-Trait Anxiety Inventory (STAI) [38]; Eysenck Personality Questionnaire (EPQ) [39]; Coping Inventory for Stressful Situations (CISS) [40]; Language and Words; Cognitive Failures Questionnaire (CFQ) [41]; WHO Quality of life BREF (WHOQOL-Bref) [42]; Cohen’s Perceived Stress Scale [43]; Positive And Negative Affect Scales (PANAS) [44]; European Quality of Life-5 Dimensions (EQ-5D-3L) [45]; Beck Depression Inventory (BDI) [46] and Work and Social Adjustment Scale (WSAS) [47].

*Brain biomarkers of potential treatment effects*

The neuroanatomical underpinnings of potential beneficial cognitive effects of CR are explored with functional and structural magnetic resonance imaging (fMRI and MRI) at baseline and weeks 12 and 26. In particular, we will investigate whether CR modulates neural responses underlying memory, executive function and social cognition with a picture encoding task (revised from Miskowiak et al [48]), an N-back (2-back versus 0-back) working memory task, and an emotional face processing task using fearful and happy Nimstim faces, respectively. We hypothesise that the CR group compared with the standard treatment group will 1) increase memory-related activation in medial temporal regions including the hippocampus and that this will correlated with memory performance 2) enhance neural response in a fronto-parietal network during the 2-back task which is associated with improved executive function, and 3) modulate neural responses to emotional faces in a fronto-limbic circuitry, which is accompanied by improved facial expression recognition. In addition, we will explore whether CR induces changes in hippocampus structure, white matter fibre structure and brain connectivity.

### Imaging data analysis

fMRI and MRI data is collected with a Siemens Trio scanner operating at 3.0 T at the Danish Research Centre for Magnetic Resonance (DRCMR), Hvidovre Hospital, Denmark. Image analysis is carried out using tools from FSL (FMRIB Software Library, <http://www.fmrib.ox.ac.uk/fsl>) [49]. BET is used for brain extraction of the T1-weighted images and FLIRT is used to perform affine alignment of the diffusion and T1-weighted images. The FIRST tool which is part of FSL (FMRIB's Software Library, [www.fmrib.ox.ac.uk/fsl](https://mail/owa/redir.aspx?C=opxaRhXWAkiFOa_nHKSnDnZ4PHS6SdAItfc9o5b-sYXQjKitijN-HQEPW04KN1fRLAmOkEgz0WI.&URL=http%3A%2F%2Fwww.fmrib.ox.ac.uk%2Ffsl) version 5.0) is used to automatically segment the hippocampus for analyses of changes in volume and shape. fMRI data from the memory and emotion processing tasks will pre-processed and analysed using FEAT (FMRIB Expert Analysis Tool) version 6.0, part of FSL, while fMRI data from the N–back task will pre-processed and analysed Statistical Parametric Mapping (SPM) software version 8. Region of interest (ROI) analyses of prefrontal, limbic and parietal regions, as well as exploratory whole-brain analyses will be performed. Diffusion images will be analysed using tools from FDT (FMRIB Diffusion Toolkit). Probabilistic modelling of diffusion parameters and tractography are carried out using previously described methods [50].

In particular, we will compare differences in neural activity from baseline to post-treatment between CR and standard treatment groups for patients who have completed both baseline and post-treatment scanning sessions. In addition, cross-sectional comparisons between the groups post-treatment will be made in the event of a missing data at baseline. Pearson’s coefficients are calculated to determine the relationship between MRI changes and neuropsychological improvements.

## Sample size and power calculation

Test sample and statistical power is calculated by nQuery Advisor 5.0 software. The primary outcome is a change in verbal memory measured with RAVLT from baseline to week 12 between CR and standard treatment groups. A recent study demonstrated that the average California Verbal Learning Test (CVLT; a verbal learning test equivalent to the RAVLT) total recall score for patients with remitted bipolar disorder is 52.0 whilst healthy controls matched on age was 60.7 (out of maximum 75) [51]. Based on this, we would expect a clinically relevant difference in the change between groups to be at least 4 points in RAVLT total recall. Assuming an average RAVLT total recall score of 56 subsequent to CR and 52 (i.e. difference in the change of 4 points) subsequent to standard treatment and a standard deviation of 4 points for both groups, a sample size of N=40 patients (N=20 per group) will achieve a statistical power of 86% to demonstrate a clinically relevant verbal memory improvement with CR versus standard treatment.

**Statistical analyses of primary, secondary and tertiary outcomes**

For investigation of the effects of CR versus standard treatment from baseline to week 12 (primary outcome assessment time) we will use repeated measures analysis of covariance (ANCOVA) with adjustment for stratification variables, and with and without adjustment for mood changes from baseline to week 12. For investigation of long-term effects of CR versus standard treatment in week 26, we will implement a linear mixed effects model with random intercept for each participant, structured as a two-level model specifying a correlation of samples within participants, with adjustment for stratification variables, and with and without adjustments for mood changes. Significant interactions will be analysed further with simple main effect analyses. All statistical analyses of behavioural data, mood ratings and questionnaires will be performed using the Statistical Package for Social Sciences (SPSS).

## Ethical considerations

The study is approved by The Regional Committee on Biomedical Research Ethics (protocol number H-1-2010-039), The Danish Data Protection Agency (protocol number 2010-41-4710) and ClinicalTrials.gov (identifier NCT01457235).

## Current trial status

Patient enrolment started in September 2011 and is ongoing until minimum 40 patients have completed assessment at week 12 (primary outcome assessment time).

**Discussion**

The present trial is the first RCT to investigate the effects of CR on cognitive function in BD. Data from patients with schizophrenia and preliminary results from small exploratory studies in mixed groups of patients with affective disorders suggest beneficial effects [16-19] although a recent study of functional remediation in BD showed no significant effect on cognition [20]. The present trial therefore serves to meet the clinical need for more specific and thorough investigations of the effects of CR on patients’ cognitive function.

*Limitations*

CR in a group setting has been investigated in a few studies only [15;20] and it is unclear whether if group therapy is less appealing than individual therapy for patients with cognitive difficulties. It is, however, our experience from the pilot study and patient feedback from the present trial that patients benefit from group based training; it is often easier for patients to acknowledge and accept their own cognitive difficulties and to learn to cope with them when they are aware of other patient’s problems and improvements. Another limitation is that it is unclear how many training sessions are optimal for bipolar patients with cognitive dysfunction. In the uncontrolled study by Deckersbach et al [18], 14 individual sessions of CR were provided and results showed lower residual symptoms and increased occupational and overall psychosocial functioning in patients with BD. Moreover, improvements in executive functioning were associated with improved occupational functioning. Studies of patients with schizophrenia have typically included an average treatment length of 32.2 hours (range=4-130), provided over 16.7 weeks (range=2-104) with a therapy intensity of 2.2 sessions per week (range=0.6-5) [15].

We chose to provide 12+1 sessions based on the experience from our pilot study that this length is sufficient for improvement in most patients. However, if this first exploratory study finds an effect of CR on cognition, future studies are warranted to explore the optimal length of treatment and frequency of CR sessions. Finally, the choice of a good control group is often complicated in psychological intervention studies. We chose to compare CR as an add-on to standard treatment with standard treatment alone. With such a design we cannot be sure that a potential beneficial effect of the active intervention is specifically due to the CR program or an unspecific effect of solely taking part in group sessions. Colom et al [52] used an elegant, however, also time and resource consuming design by comparing group psychoeducation with unspecific group intervention in their seminal paper aiming to detect the specific effects of psychoeducation. It can be argued whether if the interaction between patients in the unspecific group intervention is comparable to part of the CR treatment. Using our design, the entire combined effect of CR and group sessions, including the interaction with other patients with similar problems in-group sessions, is investigated.

### Advantages

RAVLT was chosen as the primary outcome measure for the following reasons: a) remitted patients with BD show deficits on this test [53;54]; b) it is a standardised, internationally accepted and valid test of verbal memory [55]; c) there is a high correlation between verbal memory and psychosocial function [56], which makes it clinically relevant; and d) other RCTs have found significant effects of CR on verbal memory in patients with schizophrenia [57;58;59]. However, there is also consistent evidence for trait-related deficits in sustained attention in BD and negative effects of this on psychosocial function [56]. In addition, CR has also been shown to improve attention in schizophrenia [57;59] and unipolar disorder [17], and both verbal memory and sustained attention are addressed in our CR programme. Therefore, the priority of verbal memory over sustained attention as the primary outcome may be somewhat arbitrary.

The study has a naturalistic approach with few exclusion criteria. Patients should suffer from BD, should be in complete or partial remission and should self-report cognitive difficulties. To make treatment with CR meaningful, patients with current substance or alcohol abuse or significant suicide risk are excluded but standard psychopharmacological treatment was allowed. Consequently, if CR is found to produce beneficial effects, the intervention can be implemented to treat all bipolar patients in complete or partial remission with subjective complaints of cognitive difficulties. We expect a high patient acceptance and adherence as the CR program is easy to follow, not too time consuming and due to the supportive positive effects from group setting in itself. From a feasibility perspective, the intervention is relatively inexpensive as two therapists in our affective disorder clinic deliver the group sessions. Although the CR intervention is standardised according to a manual and the computer training software [26;27] it is likely that treatment with CR is best offered in specialised affective disorder clinics with expertise within pharmacological and psychological treatment of bipolar disorder.

## Perspectives

If CR proves effective, CR can easily be implemented in future treatment of BD in order to facilitate the patients’ cognitive function and thereby potentially improve their psychosocial function.

## Competing interests

LVK has within the last three years been a consultant for Lundbeck and AstraZenica. MV has been a consultant for Eli Lilly, Lundbeck, AstraZeneca and Servier. KM has been a consultant for Lundbeck. KD and GMA have no competing interests.

## Authors’ contributions

KM, MV and LVK conceived the trial. KM authored the first draft of the trial protocol, which was then revised by LVK and MV. KD and LVK authored the first version of this article, GMA authored the CR intervention part and MV and KM revised and optimised the article.

## Acknowledgements

The study is supported by the TRYG foundation. We thank psychologists Nanna Tuxen and Anja Hubbard for their great effort in developing the CR treatment protocol, and psychiatrist Ellen Margrethe Christensen (together with GMA) for revising the CR treatment protocol and conducting the CR treatment. We also thank research assistants Anne Marie Bejerholm Svendsen, Lea Glerup, Emilie Assentoft, Nicolai Køster and Mette Marie Støttrup for their help with neurocognitive testing and fMRI scanning, and nurse Hanne Steenberg Nikolajsen and research assistant Anne Præstegaard for their help with collecting blood samples.

#### References

[1] Lopez AD, Murray CC. The global burden of disease, 1990-2020. Nat Med 1998 November;4(11):1241-3.

[2] Chamberlain SR, Sahakian BJ. Cognition in mania and depression: psychological models and clinical implications. Curr Psychiatry Rep 2004 December;6(6):451-8.

[3] Chamberlain SR, Sahakian BJ. The neuropsychology of mood disorders. Curr Psychiatry Rep 2006 December;8(6):458-63.

[4] Martinez-Aran A, Vieta E, Colom F, Reinares M, Benabarre A, Gasto C, Salamero M. Cognitive dysfunctions in bipolar disorder: evidence of neuropsychological disturbances. Psychother Psychosom 2000;69(1):2-18.

[5] Goldberg JF, Chengappa KN. Identifying and treating cognitive impairment in bipolar disorder. Bipolar Disord 2009 June;11 Suppl 2:123-37.

[6] Burdick KE, Goldberg JF, Harrow M, Faull RN, Malhotra AK. Neurocognition as a stable endophenotype in bipolar disorder and schizophrenia. J Nerv Ment Dis 2006 April;194(4):255-60.

[7] Bozikas VP, Tonia T, Fokas K, Karavatos A, Kosmidis MH. Impaired emotion processing in remitted patients with bipolar disorder. J Affect Disord 2006 March;91(1):53-6.

[8] Getz GE, Shear PK, Strakowski SM. Facial affect recognition deficits in bipolar disorder. J Int Neuropsychol Soc 2003 May;9(4):623-32.

[9] Arts B, Jabben N, Krabbendam L, van OJ. A 2-year naturalistic study on cognitive functioning in bipolar disorder. Acta Psychiatr Scand 2011 March;123(3):190-205.

[10] Lopez-Jaramillo C, Lopera-Vasquez J, Ospina-Duque J, Garcia J, Gallo A, Cortez V, Palacio C, Torrent C, Martinez-Aran A, Vieta E. Lithium treatment effects on the neuropsychological functioning of patients with bipolar I disorder. J Clin Psychiatry 2010 August;71(8):1055-60.

[11] Torrent C, Martinez-Aran A, del Mar BC, Reinares M, Daban C, Sole B, Rosa AR, Tabares-Seisdedos R, Popovic D, Salamero M, Vieta E. Long-term outcome of cognitive impairment in bipolar disorder. J Clin Psychiatry 2012 July;73(7):e899-e905.

[12] Martinez-Aran A, Vieta E, Reinares M, Colom F, Torrent C, Sanchez-Moreno J, Benabarre A, Goikolea JM, Comes M, Salamero M. Cognitive function across manic or hypomanic, depressed, and euthymic states in bipolar disorder. Am J Psychiatry 2004 February;161(2):262-70.

[13] Martino DJ, Strejilevich SA, Scapola M, Igoa A, Marengo E, Ais ED, Perinot L. Heterogeneity in cognitive functioning among patients with bipolar disorder. J Affect Disord 2008 July;109(1-2):149-56.

[14] Brissos S, Dias VV, Carita AI, Martinez-Aran A. Quality of life in bipolar type I disorder and schizophrenia in remission: clinical and neurocognitive correlates. Psychiatry Res 2008 July 15;160(1):55-62.

[15] Wykes T, Huddy V, Cellard C, McGurk SR, Czobor P. A meta-analysis of cognitive remediation for schizophrenia: methodology and effect sizes. Am J Psychiatry 2011 May;168(5):472-85.

[16] Anaya C, Martinez AA, Ayuso-Mateos JL, Wykes T, Vieta E, Scott J. A systematic review of cognitive remediation for schizo-affective and affective disorders. J Affect Disord 2012 December 15;142(1-3):13-21.

[17] Elgamal S, McKinnon MC, Ramakrishnan K, Joffe RT, MacQueen G. Successful computer-assisted cognitive remediation therapy in patients with unipolar depression: a proof of principle study. Psychol Med 2007 September;37(9):1229-38.

[18] Deckersbach T, Nierenberg AA, Kessler R, Lund HG, Ametrano RM, Sachs G, Rauch SL, Dougherty D. RESEARCH: Cognitive rehabilitation for bipolar disorder: An open trial for employed patients with residual depressive symptoms. CNS Neurosci Ther 2010 October;16(5):298-307.

[19] Naismith SL, Redoblado-Hodge MA, Lewis SJ, Scott EM, Hickie IB. Cognitive training in affective disorders improves memory: a preliminary study using the NEAR approach. J Affect Disord 2010 March;121(3):258-62.

[20] Torrent C, del Mar BC, Martinez-Aran A, Valle J, Amann BL, Gonzalez-Pinto A, Crespo JM, Ibanez A, Garcia-Portilla MP, Tabares-Seisdedos R, Arango C, Colom F, Sole B, Pacchiarotti I, Rosa AR, Ayuso-Mateos JL, Anaya C, Fernandez P, Landin-Romero R, Alonso-Lana S, Ortiz-Gil J, Segura B, Barbeito S, Vega P, Fernandez M et al. Efficacy of Functional Remediation in Bipolar Disorder: A Multicenter Randomized Controlled Study. Am J Psychiatry 2013 March 20.

[21] Wing JK, Babor T, Brugha T, Burke J, Cooper JE, Giel R, Jablenski A, Regier D, Sartorius N. SCAN. Schedules for Clinical Assessment in Neuropsychiatry. Arch Gen Psychiatry 1990 June;47(6):589-93.

[22] HAMILTON M. A rating scale for depression. J Neurol Neurosurg Psychiatry 1960 February;23:56-62.

[23] Young RC, Biggs JT, Ziegler VE, Meyer DA. A rating scale for mania: reliability, validity and sensitivity. Br J Psychiatry 1978 November;133:429-35.

[24] Fava M, Graves LM, Benazzi F, Scalia MJ, Iosifescu DV, Alpert JE, Papakostas GI. A cross-sectional study of the prevalence of cognitive and physical symptoms during long-term antidepressant treatment. J Clin Psychiatry 2006 November;67(11):1754-9.

[25] Wykes T, Reeder C, Landau S, Everitt B, Knapp M, Patel A, Romeo R. Cognitive remediation therapy in schizophrenia: randomised controlled trial. Br J Psychiatry 2007 May;190:421-7.

[26] RehaCom [computer program], Hasomed, DE. Version 6.1 2011. <http://www.hasomed.de/en/home.html>

[27] RehaCom [computer program], Hasomed, DE. Version 6.2 2012.

<http://www.hasomed.de/en/home.html>

[28] Chiesa A, Calati R, Serretti A. Does mindfulness training improve cognitive abilities? A systematic review of neuropsychological findings. Clin Psychol Rev 2011 April;31(3):449-64.

[29] Pharma Consulting Group. 2013.

Ref Type: Online Source

[30] Rey A. Psychological examination of traumatic encephalopathy. Archieves de Psychologic 1941;28:286-340.

[31] Rey A. L'examen clinique en psychologie [Clinical tests in psychology]. Paris: Presses Universitaires de France; 1964.

[32] Army Individual Test Battery. Manual of Directions and Scoring. Washington, DC: War Department, Adjutant General's Office; 1944.

[33] Randolph C. RBANS manual: Repeatable Battery for the Assessment of Neuropsychological Status. San Antonio, TX: The Psychological Corporation; 1998.

[34] Wechsler D. Wechsler Adult Intelligence Scale-III. San Antonio: The Psychological Corporation; 1997.

[35] Borkowski JG, Benton AL, Spreen O. Word fluency and brain damage. Neuropsychologia 1967;5:135-40.

[36] Nelson HE, O'Connell A. Dementia: the estimation of premorbid intelligence levels using the New Adult Reading Test. Cortex 1978 June;14(2):234-44.

[37] Rosa AR, Sanchez-Moreno J, Martinez-Aran A, Salamero M, Torrent C, Reinares M, Comes M, Colom F, Van RW, Ayuso-Mateos JL, Kapczinski F, Vieta E. Validity and reliability of the Functioning Assessment Short Test (FAST) in bipolar disorder. Clin Pract Epidemiol Ment Health 2007;3:5.

[38] Spielberger CD. State-Trait Anxiety Inventory: Bibliography. 2nd ed. Palo Alto, CA: Consulting Psychologists Press; 1989.

[39] Eysenck HJ, Eysenck SBG. Manual of the Eysenck Personality Questionnaire. London: Hodder and Stoughton; 1975.

[40] Endler NS, Parker JDA. Coping Inventory for Stressful Situations (CISS): Manual. Toronto: Multi-Health Systems; 1990.

[41] Broadbent DE, Cooper PF, FitzGerald P, Parkes KR. The Cognitive Failures Questionnaire (CFQ) and its correlates. Br J Clin Psychol 1982 February;21 (Pt 1):1-16.

[42] WHOQOL Group. The development of the World Health Organisation quality of life assessment instrument (the WHOQOL). Berlin, Heidelberg, New York: Springer; 1994.

[43] Cohen S, Kamarck T, Mermelstein R. A global measure of perceived stress. J Health Soc Behav 1983 December;24(4):385-96.

[44] Watson D, Clark LA, Tellegen A. Development and validation of brief measures of positive and negative affect: the PANAS scales. J Pers Soc Psychol 1988 June;54(6):1063-70.

[45] EuroQol--a new facility for the measurement of health-related quality of life. The EuroQol Group. Health Policy 1990 December;16(3):199-208.

[46] BECK AT, WARD CH, MENDELSON M, MOCK J, ERBAUGH J. An inventory for measuring depression. Arch Gen Psychiatry 1961 June;4:561-71.

[47] Mundt JC, Marks IM, Shear MK, Greist JH. The Work and Social Adjustment Scale: a simple measure of impairment in functioning. Br J Psychiatry 2002 May;180:461-4.

[48] Miskowiak K, O'Sullivan U, Harmer CJ. Erythropoietin enhances hippocampal response during memory retrieval in humans. J Neurosci 2007 March 14;27(11):2788-92.

[49] Smith S, Jenkinson M, Woolrich M, Beckmann C, Behrens T, Johansen-Berg H, Bannister P, De Luca M, Drobnjak I, Flitney D, Niazy R, Saunders J, Vickers J, Zhang Y, De Stefano N, Brady J, Matthews P. Advances in functional and structural MR image analysis and implementation as FSL. NeuroImage 2004 23(S1):208–219.

[50] Behrens TE, Berg HJ, Jbabdi S, Rushworth MF, Woolrich MW. Probabilistic diffusion tractography with multiple fibre orientations: What can we gain?
Neuroimage. 2007 Jan 1;34(1):144-55. Epub 2006 Oct 27.

[51] Smith DJ, Muir WJ, Blackwood DH. Neurocognitive impairment in euthymic young adults with bipolar spectrum disorder and recurrent major depressive disorder. Bipolar Disord 2006 February;8(1):40-6.

[52] Colom F, Vieta E, Martinez-Aran A, Reinares M, Goikolea JM, Benabarre A, Torrent C, Comes M, Corbella B, Parramon G, Corominas J. A randomized trial on the efficacy of group psychoeducation in the prophylaxis of recurrences in bipolar patients whose disease is in remission. Arch Gen Psychiatry 2003 April;60(4):402-7.

[53] Robinson LJ, Thompson JM, Gallagher P, Goswami U, Young AH, Ferrier IN, Moore PB. A meta-analysis of cognitive deficits in euthymic patients with bipolar disorder. J Affect Disord 2006 July;93(1-3):105-15.

[54] Robinson LJ, Ferrier IN. Evolution of cognitive impairment in bipolar disorder: a systematic review of cross-sectional evidence. Bipolar Disord 2006 April;8(2):103-16.

[55] Macartney-Filgate MS, Vriezen ER. Intercorrelation of clinical tests of verbal memory. Arch Clin Neuropsychol 1988;3(2):121-6.

[56] Depp CA, Mausbach BT, Harmell AL, Savla GN, Bowie CR, Harvey PD, Patterson TL. Meta-analysis of the association between cognitive abilities and everyday functioning in bipolar disorder. Bipolar Disord 2012 May;14(3):217-26.

[57] d'Amato T, Bation R, Cochet A, Jalenques I, Galland F, Giraud-Baro E, Pacaud-Troncin M, Augier-Astolfi F, Llorca PM, Saoud M, Brunelin J. A randomized, controlled trial of computer-assisted cognitive remediation for schizophrenia. Schizophr Res 2011 February;125(2-3):284-90.

[58] McGurk SR, Mueser KT, DeRosa TJ, Wolfe R. Work, recovery, and comorbidity in schizophrenia: a randomized controlled trial of cognitive remediation. Schizophr Bull 2009 March;35(2):319-35.

[59] Grynszpan O, Perbal S, Pelissolo A, Fossati P, Jouvent R, Dubal S, Perez-Diaz F. Efficacy and specificity of computer-assisted cognitive remediation in schizophrenia: a meta-analytical study. Psychol Med 2011 January;41(1):163-73.
